# Identification and analysis of the pigment composition and sources in the colored cocoon of the silkworm, *Bombyx mori* , by HPLC-DAD

**DOI:** 10.1093/jis/14.1.31

**Published:** 2014-01-01

**Authors:** Lin Zhu, Yu-Qing Zhang

**Affiliations:** Silk Biotechnology Laboratory, School of Basic Medical and Biological Sciences, Soochow University, No. 199, 702-2303 Room, Renai Road, Dushuhu Higher Edu. Town, Suzhou 215123, PR China

**Keywords:** *β* -carotene, chlorophyll, lutein, mulberry leaf

## Abstract

This study used the larval tissues and colored cocoons of silkworms, *Bombyx mori* L. (Lepidoptera: Bombycidae), that were fed leaves of cultivated mulberry, Husang 32, as experimental material. The pigment composition and content in colored cocoons and tissues of the 5th instar larvae and the mulberry leaves were rapidly detected using organic solvent extraction and reverse phase high-performance liquid chromatography with diode array detection. It was found that the mulberry leaf mainly contained four types of pigment: lutein (30.86%), *β* -carotene (26.3%), chlorophyll *a* (24.62%), and chlorophyll *b* (18.21%). The silk glands, blood, and cocoon shells of six yellow-red cocoons were used as the experimental materials. The results showed that there were generally two kinds of carotenoids (lutein and *β* -carotene) in the silk gland and cocoon shell, a little violaxanthin was detected in silk gland, and the pigment found in the blood was mainly lutein in all varieties of silkworm tested. Chlorophyll *a* and *b* had not been digested and utilized in the yellow-red series of silkworm. The method used to detect visible pigments reported here could be used to breed new colors of cocoons and to develop and utilize the pigments found in mulberry.

## Introduction

The awareness of environmental protection and health care consideration has increased as scientific technology and the economy have developed. The concept of using ecologically-compatible clothing is becoming more established, and returning to nature has become central to many new product developments. Naturally-colored silk has natural luster, a soft texture, and avoids the use of chemical dyeing. It has become one of the most important aspects of sericulture development and offers considerable market and developmental potential. In China, naturally-colored cocoons are mainly divided into two series: yellow-red and green. Generally speaking, the color of the green series is mainly due to the flavonoid compounds ( Tamura et al. 2002 ), but the flavonoids of mulberry leaf may need to pass through a series of chemical modifications in the silkworm, *Bombyx mori* L. (Lepidoptera: Bombycidae), such as glycosylation (Hirayama et al. 2006; Hirayama et al. 2008 ). The color of the yellow-red cocoon is due to the carotenoids found in mulberry leaves, including carotene and carrot alcohols ( Tsuchida et al. 2004 ; Bhosale et al. 2007 ).

Early in the 20th century, researchers began to study the mechanism controlling naturally-colored cocoons. The study of genetic linkage positioning showed that three genes controlled the formation of yellow-red cocoons: *Yellow blood (Y), Inhibitor gene (I)* , and *Yellow cocoon (C)* ( Doira.1992 ). In addition, carotenoids have been shown to be transported using apolipoproteins in the insect ( Suehida et al. 1998 ). Jouni and Wells (1996) extracted an apolipoprotein, lutein binding protein (LBP), from the midgut of *B. mori* . Another important apolipoprotein in *B. mori* , carotenoid binding protein (CBP), is the coding product of *Yellow blood (Y* ) and is closely associated with the absorption and transportation of carotenoids, especially lutein. Therefore, the absorption and modification of mulberry leaf pigments in *B. mori* is the key to the formation of colored cocoons. This process is affected by the genetic type of the silkworm and the carotenoid content in the midgut, blood, and silk gland. These factors determine the color of the cocoons ( Sakudoh et al. 2010 ).

Although the genetic characteristics and the formation of colored cocoons are now well understood, there is still no fast, systematic method to detect the composition and content of the pigments in colored cocoons, silkworm tissues, and mulberry leaves. This has made it difficult to analyze the pigments in silkworms and to breed differentiated strains of silkworm.

Lang (2009) investigated the differences between the pigments, metabolism, and the inheritance mechanisms in different-colored cocoon varieties. The pigment content and types between different-colored cocoons from a number of silkworm varieties was analyzed by using ethanol to extract the pigments in cocoons and the ternary linear gradient system of reversed-phase high-performance liquid chromatography to detect the pigment content. The results showed that carotenoids and flavonoids were the major pigments in the yellow-red and green cocoon series, respectively ( Lang 2009 ). Other research has shown that the carotenoid content in silkworms affects cocoon color differentiation ( Niu 2010 ). Tang et al. (2011) extracted the carotenoids using hexane:ethanol:acetone (2:1:1), and then the low-phase was extracted again with ether. The detection method used to study the carotenoids in mulberry leaves and yellow-red cocoons was the ternary linear gradient system of RP-HPLC. These studies showed that mulberry leaves and cocoons contained four major carotenoids: violaxanthin, lutein, *α* -carotene, and *β* -carotene. The accumulation pattern of the coloration pigments in the midgut, blood, and silk gland of all the developmental stages in 5th instar larvae for four types of cocoon color and eight strains of silkworm showed that as age increased, the carotenoid accumulation order for six silkworm varieties, with regards to the midgut and the blood, was lutein > violaxanthin > *β* -carotene > *α* -carotene, and the silk gland was found to favor the accumulation of certain carotenoids. Xu et al. (2010) analyzed the metabolic changes to pigments in silkworm. The results showed that the flavonoids accumulated in the midgut of the 5th instar larvae and carotenoids accumulated in the silk gland. Cocoon color was found to be closely-related to the content of two kinds of pigments in the silk gland. They had come from mulberry leaves, were digested and absorbed in the midgut, and then were transported through the blood into the silk gland to produce a cumulative effect.

In this study, we used rapid extraction by organic solvents and reversed-phase high-performance liquid chromatography with diode array detection (HPLC-DAD) to efficiently, rapidly, and accurately identify and analyze the pigment composition and contents in mulberry leaves and naturally-colored cocoons. The method provided reliable data that can be used to cultivate new varieties of silkworm that have different-colored cocoons.

## Materials and Methods

### Materials

The yellow-red series cocoons, the 5th instar larvae, and the mulberry leaves (cultivar Husang 32) were provided by the Sericulture Department at Soochow University, China. The tissues (blood and silk glands) and the mulberry leaves were collected and immediately turned into powder through freeze-drying. Lutein was purchased from Shanghai Medical Technology Co. ( www.pharm-sh.com.cn ); the violaxanthin was purchased from CaroteNature ( www.carotenature.com ); *β* -carotene and chlorophyll *a* were purchased from Wako Pure Chemical Industries ( www.wako-chem.co.jp ) and chlorophyll *b* was purchased from B & K Technology Group ( www.tina0504.en.busytrade.com ).

### Extraction of Pigments

Different organic solvents were used to optimize the extraction of pigments from the colored cocoons. Exactly 0.2 g of colored cocoon was shredded, and the organic solvents were then used to extract the compounds. The extract was then centrifuged (140,000 rpm) and filtered prior to analyzing by HPLC. To extract the pigments from the blood and silk gland, blood lyophilized powder was dissolved in 70% ethanol (10.0 mg/mL) and incubated at 60°C for one hour, then centrifuged at 140,000 rpm (Avanti J-30I centrifuge; Beckman Coulter, www.beckmancoulter.com ) for 15 min to obtain the supernatant. The silk gland lyophilized powder was dissolved in acetone and incubated at 60°C and then centrifuged as outlined above. In order to extract the pigments from the the mulberry leaves, the lyophilized powder was ground in acetone three times and then centrifuged at 140,000 rpm to obtain the supernatant.

### Standard samples

The standard samples were dissolved in acetone so that exact concentration solutions could be created. These were then stored at -20°C. Five different standard sample solutions were combined to make a mixed solution for HPLC analysis. Injection volumes were 2.5 µL, 5 µL, 10 µL, 15 µL, and 20 µL, and each gradient was repeated three times.

### HPLC analytical conditions

A previously-reported method ( Kidmose 2001 ) was slightly modified for HPLC analysis. The HPLC system consisted of an autosampler, a pump (LC-20AT), a DAD detector (SPD-M20A), and a VP-ODS column (SHIMADZU 250 ×4.6 mm, www.shimadzu.com ). The pigments were analyzed using a mobile phase consisting of (A) 80% methanol and (B) 100% ethyl acetate, from 0 to 2.5 min and then eluted with gradient A:B (80:20–77.5:22.5) from 2.5 to 17.5 min; gradient A:B (77.5:22.5–50:50) from 17.5 to 20 min; isocratic A:B (50:50) from 20 to 21.5 min; gradient A:B (50:50– 20:80) from 21.5 to 23.5min; isocratic A:B (20:80) from 23.5 to 28.5 min; gradient A:B (20:80–0:100) and isocratic A:B (0:100) from 28.5 to 40 min. The flow rate was 1 mL/min, and eluate absorbance was monitored at 440 nm using a scanning range of 200 nm to 800 nm.

### Colored cocoons for UV-visible spectroscopy

Thirty-two yellow-red cocoons were cut to 2 cm ^2^ in size so that they could be scanned by an integrating sphere (HSP6000, www.hopoo.lightstrade.com ) using a white cocoon as the control and with a wavelength range between 260 nm and 700 nm.

## Results

### Standard curve

**Figure 1.**
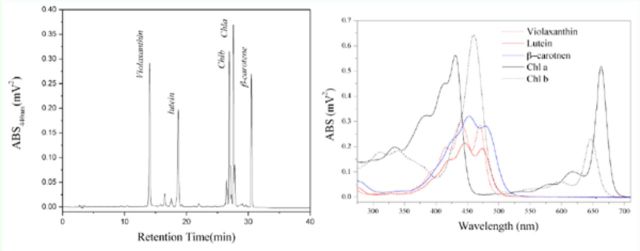
Chromatogram and UV-visible spectra of five standard samples by HPLC-DAD. High quality figures are available online.

**Figure 2.**
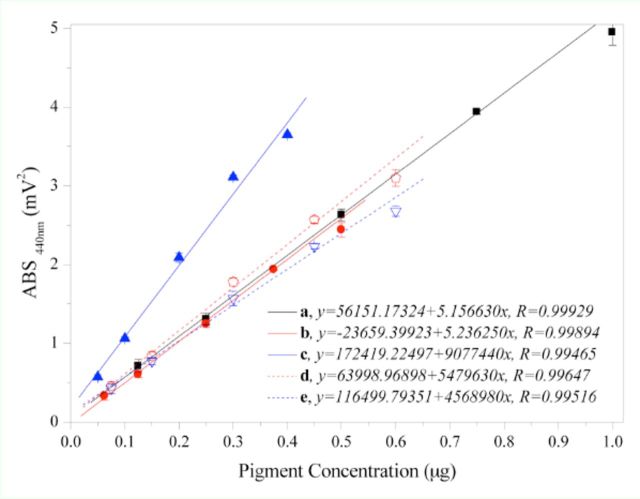
Standard curves of five standard samples. a: lutein, b: *β* -carotene, c:violaxanthin , d: chlorophyll *a* , e: chlorophyll *b* . The data in the figure are average values (±SD) of three repeated measurements. High quality figures are available online.

Figure 1 shows the chromatogram for the standard samples and their UV-visible spectra ( Figure 1 ). Taking the concentration of a standard sample as Y and the chromatographic peak area as X, it is possible to draw a standard curve ( Figure 2 ).

The linear regression equations for the five standard samples were as follows: violaxanthin, y = 172419.22497 + 9077440x, R = 0.99465; lutein, y = 56151.17324 + 5156630x, R = 0.99929; chlorophyll *b* , y = 116499.79351

+ 4568980x, R = 0.99516; chlorophyll *a* , y = 63998.96898 + 5479630x, R = 0.99647, and *β* -carotene, y = -23659.39923 + 5236250x, R = 0.99894. The concentrations and the area under the chromatographic peak showed a good linear relationship.

### Carotenoid extraction from the colored cocoon shell

The total carotenoid content and two individual pigment contents were calculated according to the equation derived from the standard curve. The results are shown in Table 1 . Table 1 shows that acetone, ethyl acetate, and *n* -hexane were all able to extract carotenoids. The *n* -hexane alone could not effectively extract lutein when it was mixed in with acetone. Ethyl acetate could enhance the solubility of the non-polar carotenoids, but overall, acetone effectively extracted lutein and *β* -carotene, so this investigation used acetone to extract the various carotenoids in the cocoon.

**Table 1.**
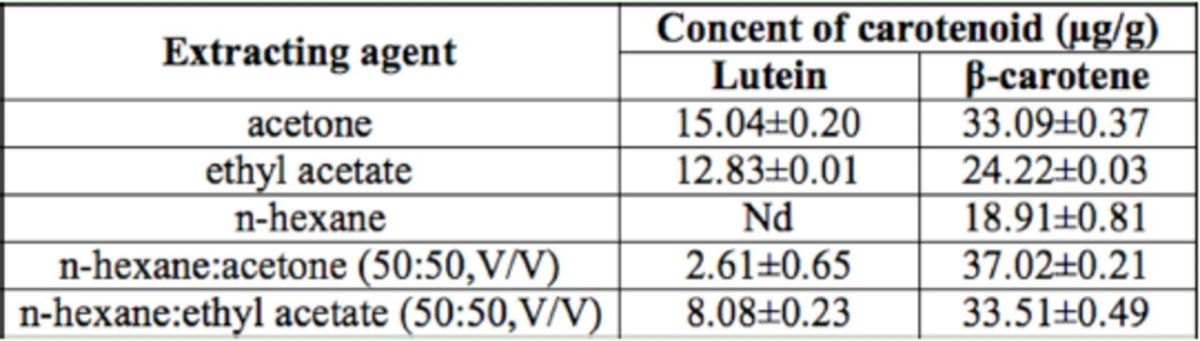
Composition and content of the carotenoids in colored cocoon shells of *Bombyx mori* extracted by different organic solvents. The data in the table are average values (±SD) of three-repeated measurements. Carotenoids analysis in tissues of yellow-red cocoons

Nd = not determined.

### Composition and contents of the pigments found in mulberry leaves

**Figure 3.**
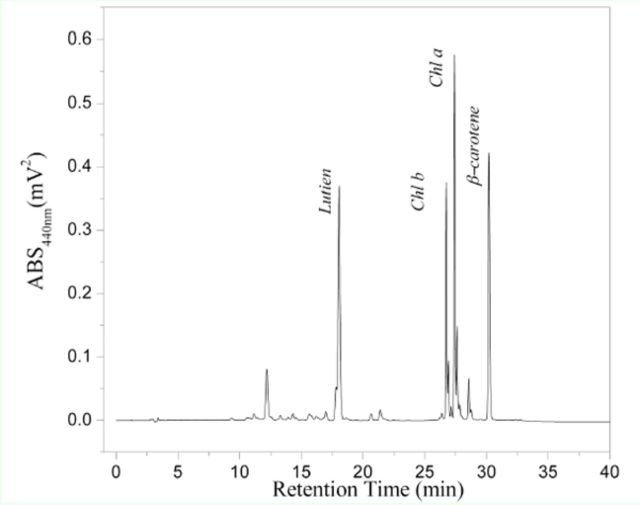
Chromatogram of pigments in mulberry leaves by HPLCDAD. High quality figures are available online.

**Figure 4.**
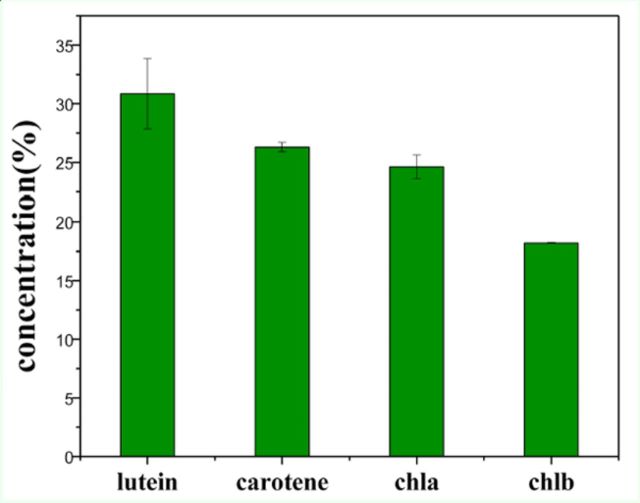
Composition and content of the pigments in mulberry leaves (leaf position was 5 or 6).The data in the figure are average values (±SD) of three repeated measurements. High quality figures are available online.

The mulberry leaves used in this investigation came from the most common variety (Husang 32), and the leaf samples were taken from leaf position 5 or 6. The pigments in the leaves were extracted with acetone and detected using RP-HPLC, which produced the chromatogram shown in Figure 3 . After comparing the retention time and the UV-visible spectrum to the standard samples, the four main peaks shown in Figure 3 were identified as: lutein (RT = 18.05867 min), chlorophyll *b* (RT = 26.74133 min), chlorophyll *a* (RT = 27.41333 min), and *β* -carotene (RT = 30.19733 min). The above four pigments in mulberry leaves were the main pigments found, and the concentrations of the pigments are shown in Figure 4 . The lutein concentration was highest (30.86%), followed by *β* -carotene (26.3%), chlorophyll *a* (24.62%), and chlorophyll *b* (18.21%).

### Carotenoids analysis in tissues of yellowred cocoons

**Figure 5.**
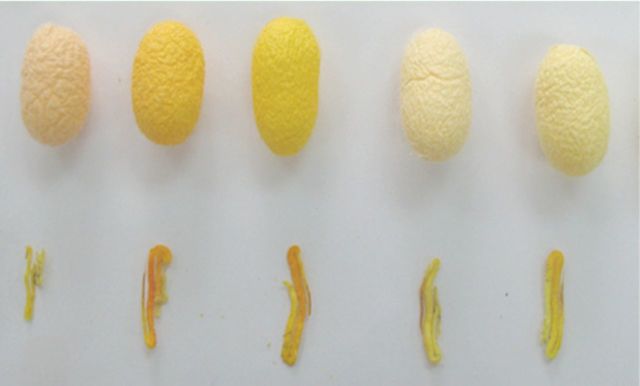
Silk glands of 5th instar larvae or *Bombyx mori* and the corresponding cocoon shells. From left to right, the kinds of coloredcocoon numbers for 1 to 5. High quality figures are available online.

**Figure 6.**
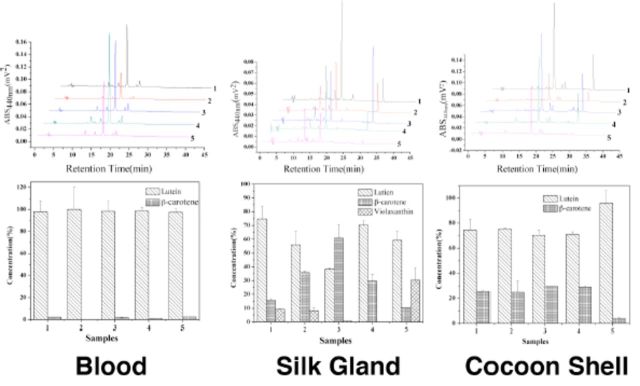
Composition and content of the pigments in mulberry leaves (leaf position was 5 or 6).The data in the figure are average values (±SD) of three repeated measurements. High quality figures are available online.

Five varieties of yellow-red cocoon shells and their corresponding blood and silk glands and cartenoid concentrations ( Figure 5 ) were measured. Figure 6 shows chromatograms for the three tissues and histograms, which show variations in carotenoid concentrations between the tissues. Only lutein and *β* -carotene were detected in the cocoon shells and blood, but in the silk gland, in addition to the above two pigments, a small amount of a third pigment, violaxanthin, was also detected. This may be because violaxanthin was the product produced when lutein and *β* -carotene were modified in the silk glands. This needs further investigating.

The relative percentage for carotenoids in the tissues and cocoon shells are shown in Table 2 . Lutein content was highest and close to 100% in the blood regardless of variety, but in the silk glands and cocoon shells there were three and two kinds of pigments, respectively. The silk glands, in particular Variety 3, contained the most *β* -carotene content, and Variety 1 contained the most lutein. In the cocoon shells, lutein was the main pigment regardless of variety. This showed that the silk gland wall was the tissue where different carotenoids were selectively absorbed in the silk gland, and that this then influenced the color of the cocoons. The results that were obtained and the color that was usually observed were different, which indicated that there were other factors affecting cocoon color. The types of carotenoids present in the yellow-red cocoon series were not significantly different, but the content and relative proportions of each showed clear significant differences.

**Table 2.**
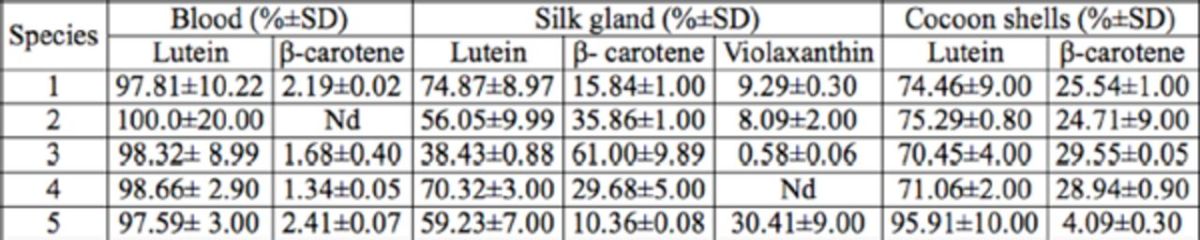
The percentage composition of carotenoids in tissues of *Bombyx mori* . The data in the table are average values (±SD) of three repeated measurements.

Nd = not determined.

### UV-visible spectroscopy of the colored cocoon surface

**Figure 7.**
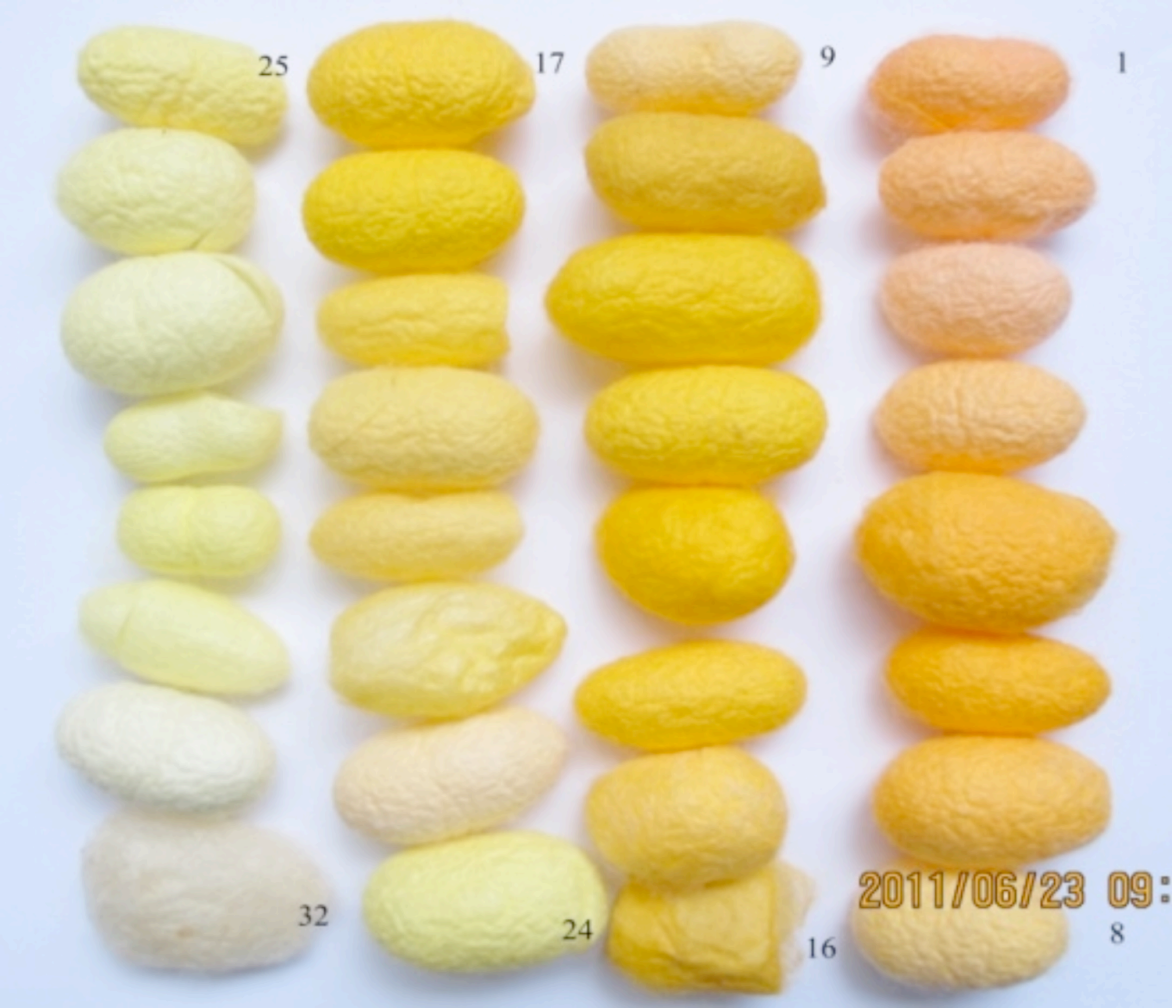
Thirty-two kinds of coloredcocoons of *Bombyx mori* . High quality figures are available online.

**Figure 8.**
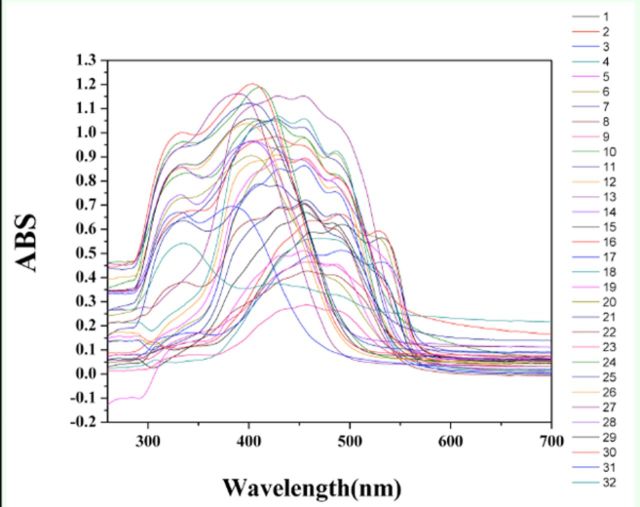
UV-visible spectra of 32 kinds of colored cocoons of *Bombyx mori* scanned by an integrating sphere High quality figures are available online.

Thirty-two yellow-red cocoons are shown in Figure 7 . The results ( Figure 8 ) indicate that the color of the cocoon gradually turned from red to yellow to green. The absorption spectrum shows a distinct change. The main absorption peak gradually shifted from 400 nm to 500 nm to 300 nm to 400 nm. As mentioned above, flavonoids are thought to affect the color of the green cocoon series, so the reason for the change may be that the carotenoid content dropped compared to the flavonoids. This would indicate that concentrations of the primary pigments and/or the ratio of different pigments have an important effect on cocoon color.

## Discussion

Mulberry leaves are the main food source for silkworms, which means that they are also the main source of the pigments that determine cocoon color. In this study, the types and contents of the pigments in mulberry leaves were analyzed by HPLC. The results showed that they contained four common pigments: lutein, *β* -carotene, chlorophyll *a* , and chlorophyll *b* . Then, the cocoon shells and the tissues of five varieties from the yellow-red cocoon series were analyzed for pigment type and content. Only the carotinoid found in the mulberry leaves appeared in the cocoons and tissues. This showed that the carotenoids in the mulberry leaves were absorbed and transported in silkworms and led to colored cocoons. However, chlorophyll *a* and *b* were not found in the cocoons. There were two main kinds of carotenoids (lutein, *β* -carotene) in the silk gland and cocoon shell, a small amount of violaxanthin was detected in the silk gland, and lutein was the main pigment in the blood. The results suggest that the silk gland wall was the main barrier that selectively absorbed different pigments. This differential absorption was the primary reason for the different-colored cocoons. The types of pigments found in the different-colored cocoons were similar, but the content and relative proportions of each pigment were significantly different.

Tang (2011) performed an in-depth investigation into the accumulation of pigments in yellow-red cocoons, using a mixture of polar and non-polar solvents to extract all the carotenoids and using a ternary gradient elution system in the RP-HPLC in order to analyze the species and contents and identify the various carotenoids present at the 5th instar larval stage ( Tang 2011 ).The purpose of our experiment was not only to precisely determine the contents of the pigments in the cocoon shells, but also to demonstrate the use of simple and rapid methods to extract and measure the pigments found in different-colored cocoons. Percentage composition data were obtained for the first time, which will have many production applications.

Thirty-two yellow-red cocoons were scanned by an integrating sphere, which clearly showed a shift in the optical absorption peak. This result can be best explained by changes in the flavonoid content in the cocoons. Studies have shown that the pigments in colored cocoons consist of flavonoids and carotenoids, and in the green cocoons the content of flavonoids is greater than the carotenoid content. A small number of other flavonoids and carotenoids have also been found ( Xu et al. 2010 ), but the flavonoids came from mulberry leaves, and the absorption and metabolism of flavonoids in male and female silkworms were different, so fluorescent cocoon sex identification was undertaken ( Zhang et al. 2010 ). The importance of flavonoids suggests that further research into the type and content of flavonoids in different-colored cocoons and its influence on color is needed.

## Abbreviations

HPLC-DAD: high-performance liquid chromatography with diode array detection

## References

[R1] Bhosale P Bernstein PS. 2007 . Vertebrate and invertebrate carotenoid-binding proteins . Arch Biochem Biophys 458 : 121 – 127 17188641 10.1016/j.abb.2006.10.005 PMC1831825

[R2] Doira H . 1992 . Genetical stocks and mutations of Bombyx mori: important genetic resources. Linkage maps and list of genetical stocks maintained in Kyushu University . Institute of Genetic Resources, Kyushu University , Japan .

[R3] Hirayama C Ono H Tamura Y Konno K Nakamura M . 2008 . Regioselective formation of quercetin 5-O-glucoside from orally administered quercetin in the silkworm. *Bombyx mori* . Phytochemistry 69 : 1141 – 1149 . 18164738 10.1016/j.phytochem.2007.11.009

[R4] Hirayama C Ono H Tamura Y Nakamura M . 2006 . C-prolinylquercetins from the yellow cocoon shell of the silkworm *Bombyx mori* . Phytochemistry 67 : 579 – 583 . 16430932 10.1016/j.phytochem.2005.11.030

[R5] Jouni ZE Wells MA . 1996 . Purification and partial characterization of a lutein-binding protein from the midgut of the silkworm *Bombyx mori* . J Biol Chem 271 : 14722 – 14726 . 8663050 10.1074/jbc.271.25.14722

[R6] Kidmose U Knuthsen P Edelenbos M Justesen U Hegelund R . 2001 . Carotenoids and flavonoids in organically grown spinach ( *Spinacia oleracea* L) genotypes after deep frozen storage . J Sci Food Agric 81 : 918 – 923 .

[R7] Lang H . 2009 . Study on the pigment of natural colored cocoon of silkworm, Bombyx mori . Soochow University , China .

[R8] Niu Y . 2010 . Structure and expression pattern analysis of CBP gene with carotenoids difference in natural coloredcocoon strains of Bombyx mori . Soochow University , China .

[R9] Sakudoh T Iizuka T Narukawa J Sezutsu H Kobayashi I Kuwazaki S Banno Y et al . 2010 . A CD36-related transmembrane protein is coordinated with an intracellular lipid-binding protein in selective carotenoid transport for cocoon coloration . Biol Chem 285 : 739 – 7751 . 10.1074/jbc.M109.074435 PMC2844218 20053988

[R10] Suehida KT Arai M Tanaka Y et al . 1998 . Lipid transfer particle catalyzes transfer of carotenoid between lipopllorins of *Bombyx mori* . Insect Biochem Mol Biol 28 : 927 – 934 . 9887509 10.1016/s0965-1748(98)00036-8

[R11] Tamura Y Nakajima K Nagayasu K Takabayashi C . 2002 . Flavonoid 5-glucosides from the cocoon shell of the silkworm. *Bombyx mori* . Phytochemistry 59 : 275 – 278 . 11830135 10.1016/s0031-9422(01)00477-0

[R12] Tang H . 2011 . The main pigment accumulation pattern of different yellow-red cocoons strains in Bombyx mori . Southwest University , China .

[R13] Tsuchida K Katagiri C Tanaka Y Tabunoki H Sato R Maekawa H Takada N Banno Y Fujii H Wells MA Jouni ZE . 2004 . The basis for colorless hemolymph and cocoons in the Y-gene recessive *Bombyx mori* mutants: a defect in the cellular uptake of carotenoids . J Insect Physiol 50 : 975 – 983 . 15518665 10.1016/j.jinsphys.2004.08.001

[R14] Xu Y Wang Y Lin J Zhong Y Lin B . 2010 . Metabolic variations of the main pigment substances in silkworm larvae spinning colored cocoons . Sci Sericulture 36 : 0619 – 0624 .

[R15] Zhang YQ Yu XH Shen WD Ma YL Zhou LX Xu NX Yi SQ. 2010 . Mechanism of fluorescent cocoon sex identification for silkworms *Bombyx mori* . Sci China Life Sci 53 : 1330 – 1339 . 21046325 10.1007/s11427-010-4084-3

